# Ecological Dynamics of Volatile Organic Compound–Mediated Interactions in *Aristolochia Contorta* With Parasitoids and Herbivores

**DOI:** 10.1002/ece3.71175

**Published:** 2025-04-02

**Authors:** Si‐Hyun Park, Jae Yeon Jang, Hangah Lim, Sang‐Gyu Kim, Jae Geun Kim

**Affiliations:** ^1^ Center for Education Research Seoul National University Seoul Republic of Korea; ^2^ Department of Biology Education Seoul National University Seoul Republic of Korea; ^3^ Department of Biological Sciences KAIST Daejeon Republic of Korea; ^4^ Department of Science Education Graduate School, Seoul National University Seoul 08826 Republic of Korea

**Keywords:** damage‐induced plant volatiles (DIPVs), defense strategy, herbivore, *Ooencyrtus* spp., *Sericinus montela*, VOCs

## Abstract

In the evolutionary arms race between plants and herbivores, sophisticated mechanisms of indirect plant defense play a pivotal role. This study investigated the role of volatile organic compounds (VOCs) in attracting the parasitoid *Ooencyrtus* spp. to *Aristolochia contorta*, while also providing insights into the interactions among *A. contorta* , the herbivore *Sericinus montela*, and *Ooencyrtus* spp. in a tritrophic context. This study utilized field surveys, olfactometer experiments, and Gas Chromatography–Mass Spectrometry (GC–MS) analysis to investigate the role of VOCs. Field surveys showed a 54.6% egg parasitism rate, with quadrats containing *A. contorta* and larvae attracting more *Ooencyrtus* spp. than those with the plant alone. In olfactometer bioassays, *Ooencyrtus* spp. preferred leaves damaged by a pattern wheel simulating herbivore damage, with 46.8% choosing these leaves over undamaged controls. Leaves treated with larval saliva were similarly attractive, drawing in 48.7% of *Ooencyrtus* spp.; however, the difference in attraction between saliva‐treated and untreated leaves was not statistically significant, suggesting saliva may not be central to *Ooencyrtus* spp. attraction. GC–MS analysis identified VOCs in damaged leaves, including hexyl acetate, cyclohexene, δ‐cadinene, α‐pinene, and β‐caryophyllene, while saliva‐treated leaves showed minimal amounts of exo‐isocitral and β‐pinene. Despite complex responses, our analysis suggests these saliva‐induced compounds do not significantly boost *Ooencyrtus* spp. attraction. This finding implies that while the VOC response to damage and saliva application is multifaceted, serving multiple defensive functions, the amount of these saliva‐induced compounds may be insufficient to substantially influence the behavior of *Ooencyrtus* spp. toward damaged leaves. Our results emphasize the role of VOCs in *A. contorta* 's indirect defense mechanisms and contribute to understanding the ecological dynamics within plant‐parasitoid‐herbivore interactions. Moreover, our findings suggest new avenues for exploring the ecological and evolutionary roles of chemical signals, highlighting the complex interactions facilitated by these cues in plant defenses.

## Graphical abstract

This study examined the role of volatile organic compounds (VOCs) in the indirect defense mechanisms of *Aristolochia contorta* against herbivores and parasitoids, utilizing field surveys, olfactometer experiments, and Gas Chromatography–Mass Spectrometry. The findings demonstrated that the VOCs emitted from damage alone were sufficient to attract the parasitoid *Ooencyrtus* spp., despite the additional but insignificant impact of compounds induced by larval saliva.

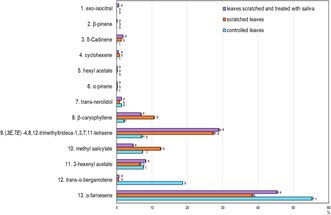

## 1 Introduction

The evolutionary arms race between plants and herbivorous insects has led to the development of sophisticated plant defense strategies, reflecting millions of years of dynamic adaptation and counter‐adaptation (Mathur et al. 2024; Whitehill et al. 2023). This ongoing struggle has driven plants to evolve a complex array of mechanisms aimed at deterring insect attacks and minimizing damage (Yactayo‐Chang et al. 2020). These strategies evolve in response to the changing tactics of herbivores, which in turn develop adaptations to overcome plant defenses, creating a perpetual cycle of interaction and counteraction (War et al. 2018).

Plants employ both direct and indirect defense mechanisms. Direct defense mechanisms in plants include physical barriers, like thick leaves or spines, and chemical defenses such as bitter‐tasting compounds or toxins (Mahawer et al. 2022; Wari et al. 2022). However, some herbivores have evolved resistance to these defenses (Wojda et al. 2020); plants have adopted indirect strategies like emitting volatile organic compounds (VOCs). These include damage‐induced plant volatiles (DIPVs, volatiles released in response to mechanical damage) and herbivore‐induced plant volatiles (HIPVs, volatiles emitted in response to herbivore attack) (Kaplan 2012; Pearse et al. 2020; Pérez‐Hedo et al. 2021). These VOCs are essential in attracting natural predators and parasitoids of herbivores, thus indirectly controlling herbivore populations and reducing herbivory (Davidson‐Lowe and Ali 2021). By alerting neighboring plants to potential threats, this signaling system prompts them to bolster their own defenses, fostering a network of plant communication that contributes to ecological balance (Gebreziher 2020; Hu et al. 2021; Paudel Timilsena et al. 2020).

Egg parasitoids, such as those from the genus *Ooencyrtus*, utilize a combination of visual, olfactory, and chemical cues to locate suitable hosts. VOCs emitted by plants in response to herbivory, including larval feeding, can serve as attractants for parasitoids (Kaplan 2012). Additionally, egg deposition on plant surfaces may induce specific VOC emissions or changes in plant surface chemistry that attract parasitoids (Fatouros et al. 2020; Hilker and Meiners 2010). These cues can enhance parasitoid host‐location efficiency, contributing to the effectiveness of indirect plant defenses.

*Ooencyrtus* spp., a genus of parasitic wasps, plays a key role in plant defense by parasitizing the eggs of various herbivorous insects across multiple orders, including Lepidoptera, Hemiptera, Coleoptera, Orthoptera, and Diptera (Fatouros et al. 2020; Ganjisaffar and Perring 2020). Their larvae consume the host egg content, effectively reducing the population of the next generation of herbivores (Conti et al. 2021; Lesieur and Farinha 2021). This biological control agent operates within a complex web of interactions, where plants, herbivores, and parasitoids are entwined in a mutualistic relationship (Ferreira and Musumeci 2021; Power et al. 2021; Rondoni et al. 2021; Sehrawat et al. 2022). The presence of *Ooencyrtus* spp. in an ecosystem can significantly enhance plant survival and fitness by mitigating the impact of herbivorous insects (Power et al. 2021).

Despite their ecological significance, little is known about the interactions between *Ooencyrtus* spp., *Aristolochia contorta* (Aristolochiaceae), and its specialist herbivore, *Sericinus montela* (Papilionidae). *S. montela*, commonly known as the dragon swallowtail, is a vulnerable species in South Korea, while *A. contorta* , its host plant, produces Aristolochic acid, a potent chemical deterrent against most herbivores (Park et al. 2023). However, *S. montela* larvae have evolved a detoxification mechanism that neutralizes Aristolochic acid, rendering this direct chemical defense ineffective (Jeong et al. 2023; Palma‐Onetto et al. 2024). This interaction exemplifies an extreme case of evolutionary specialization, where a plant's chemical defense is entirely bypassed by its specialist herbivore. Unlike many plant‐insect systems where chemical deterrence effectively reduces herbivory, *A. contorta* faces the challenge of a specialist herbivore that can overcome its toxic defenses. This evolutionary arms race has likely driven *A. contorta* to rely more heavily on indirect defenses, such as parasitoid recruitment through VOC emissions.

Furthermore, existing studies have widely considered herbivore‐induced plant volatiles (HIPVs) as key mediators of tritrophic interactions, facilitating natural enemy recruitment (Gebreziher 2020; Heil 2004; Park and Kim 2023; Poelman et al. 2009; Shivaramu et al. 2017). However, in chemically specialized plants like *A. contorta* , VOC signaling may function differently from conventional models. *S. montela* has evolved mechanisms to bypass *A. contorta* 's primary chemical defense, aristolochic acid (Jeong et al. 2023; Palma‐Onetto et al. 2024), potentially pressuring the plant to shift toward more effective indirect defense strategies. Notably, how VOCs operate in attracting parasitoids within this system remains largely unexplored. Understanding whether *A. contorta* 's VOC emissions align with established tritrophic interaction models or represent an alternative defensive strategy is crucial (Gardner and Agrawal 2002; Park and Kim 2024; War and Sharma 2014; War et al. 2018).

To address this gap, we hypothesize that VOCs emitted in response to herbivory, particularly from larval feeding, create signals that attract parasitoids like *Ooencyrtus* spp., thereby enhancing their efficiency in locating hosts. Furthermore, we specifically investigate whether mechanical damage alone (DIPVs) is sufficient to attract parasitoids or if larval saliva (HIPVs) provides additional attraction cues. Given the widespread use of saliva treatments in tritrophic interaction studies, our findings offer critical insights into the relative efficacy of these two experimental approaches and the broader implications for parasitoid recruitment in natural settings. We propose that the distribution and behavior of *Ooencyrtus* spp. in environments where *A. contorta* and *S. montela* coexist are influenced by multiple cues, including egg deposition, larval feeding, and mechanical damage. By integrating field surveys, olfactometer bioassays, and VOC analysis, our study aims to elucidate the role of these cues in mediating complex ecological interactions within this plant‐herbivore‐parasitoid system. Investigating these three interconnected aspects will contribute to our understanding of the ecological significance of VOCs in natural herbivore‐parasitoid systems and provide valuable insights into the dynamics of plant‐insect interactions, highlighting the evolutionary ingenuity of nature in developing survival strategies (Bashir et al. 2023; Thompson et al. 2022).

## 2 Methods

### 2.1 Field Survey

**FIGURE 1:**
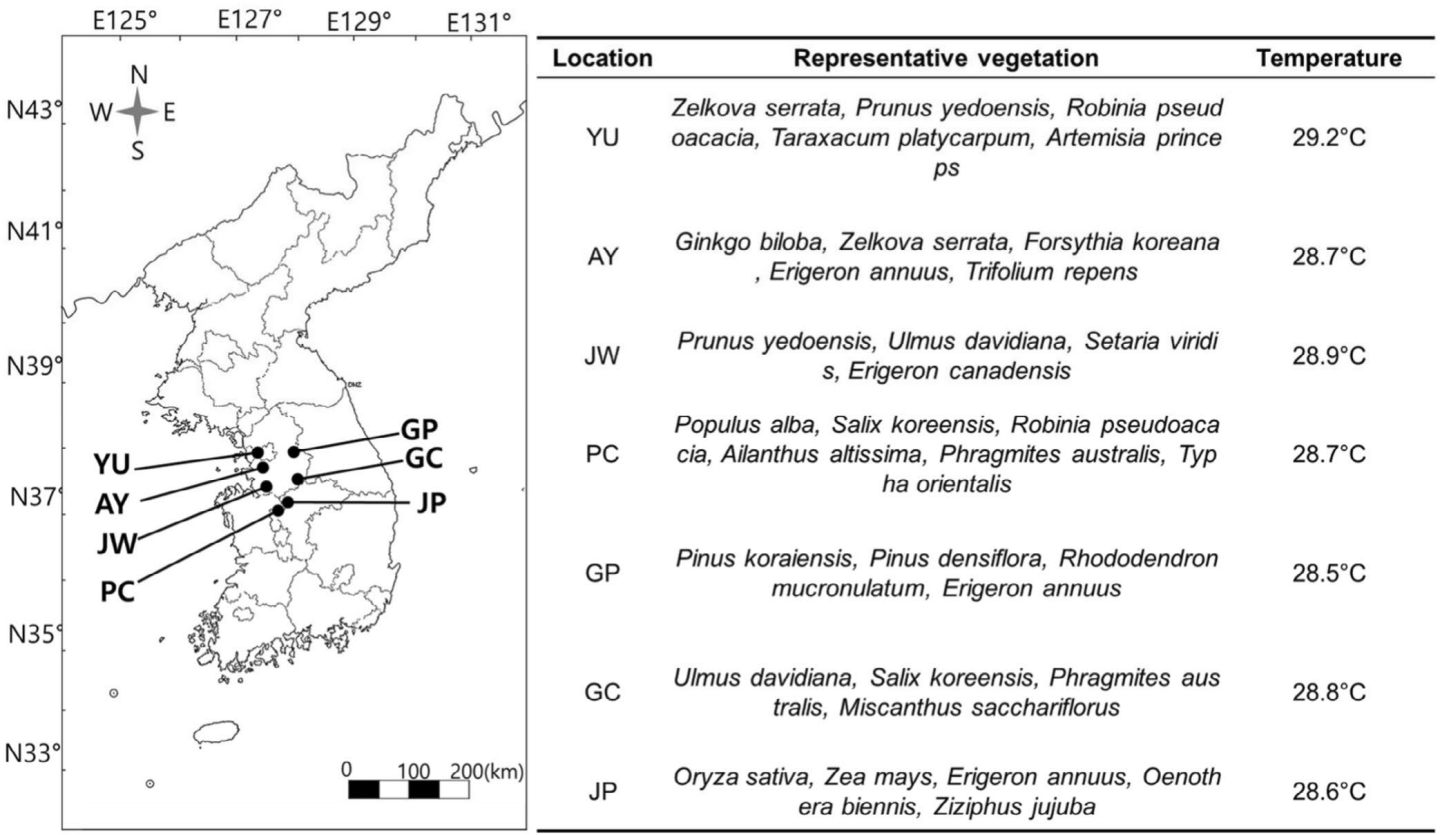
Study sites (filled circles) in South Korea with representative vegetation and temperature. AY, Anyang; GC, Gangcheon; GP, Gapyeong; JP, Jeungpyeong; JW, Jinwee; PC, Pyungchon; YU, Yeouido.

To investigate the habitats of *A. contorta* , we conducted a comprehensive review of literature and media reports (Park et al. 2019; YonhapNewsAgency 2021). This review helped us select 7 sites located on roadsides near rivers or rice paddies across South Korea for our study (Figure 1). The sites, labeled as YU (Yeouido), AY (Anyang), JW (Jinwee), PC (Pyungchon), GP (Gapyeong), GC (Gangcheon), and JP (Jeungpyeong), each had unique representative vegetation, with an average temperature of 28.8°C (Figure 1). Field surveys and observations at these sites were initiated in August 2021. We set up a total of 64 quadrats (1 m × 1 m) in the 7 different sites, randomly distributing them within each site based on the presence of *A. contorta* alone, *A. contorta* with eggs of *S. montela*, and *A. contorta* with both eggs and larvae of *S. montela*. We conducted specific searches for egg clusters, surveyed the number of eggs per cluster, noted the height at which *S. montela* egg clusters were located on *A. contorta* , and determined parasitism by inspecting egg clusters for the presence of *Ooencyrtus* spp. emergence holes or parasitoid pupae. We then calculated parasitism rates for each region. To confirm parasitism accurately, we examined each *S. montela* egg cluster for emergence holes, finding none in our survey. Afterward, we collected and monitored the eggs in a controlled lab setting, allowing us to observe directly whether *Ooencyrtus* spp. emerged, confirming the parasitism status of each cluster.

Additionally, we installed one sticky trap (35 cm x 25 cm) per quadrat, resulting in a total of 64 traps across the seven study sites. The traps were installed at the end of each field survey and positioned at a height of 1.5 m within the middle of the quadrats, at the height where egg clusters of the vine were most densely distributed. The traps were left hanging for 48 h in order to count the number of *Ooencyrtus* spp. in each condition, and after this period, we checked to ensure no new eggs had been laid. Dr. Heung‐sik Lee, a Korean entomologist from the Animal and Plant Quarantine Agency, assisted us in identifying insects.

### 2.2 Olfactometer Bioassay

We used a T‐shaped olfactometer to study the distribution preferences of *Ooencyrtus* spp., offering them different dual choices. The experiments were conducted using a T‐shaped olfactometer made of transparent glass, with a diameter of 3 cm, a stem of 30 cm, and arms of 15 cm each. Filtered air, at a rate of 0.3 L/min, was drawn through the olfactometer by a pump connected to a flowmeter. *A. contorta* seeds, collected from AY in November 2021, were cultivated in a greenhouse at Seoul National University in 2022. By August, leaves had been harvested and were subsequently divided into three treatment groups: untreated (control), scratched with a pattern wheel (mechanical damage to standardize wounding), and scratched with a pattern wheel followed by the application of *S. montela*'s saliva (collected from larvae to simulate herbivory‐induced responses). For this simulation, 20 μL of *S. montela* saliva, diluted 20 times with deionized water, was applied per wounded leaf. The saliva was collected directly from third‐instar larvae or older using pipette tips, gently stroked near the mouthparts to induce secretion. After saliva application, leaves were used in the experiment within 1 h, allowing sufficient time for defense response induction (Halitschke et al. 2001; McGale et al. 2018; Ohnmeiss and Baldwin 1994).

In the first T‐shaped olfactometer setup, the eggs were carefully removed from leaves using forceps and placed on slide glass for the experiment, with 50 fresh eggs (newly collected, pale yellow, undried, and not parasitized) of *S. montela* positioned at one end and 50 fresh eggs of *Paracycnotrachelus chinensis* (collected by gathering rolled leaves from *Quercus aliena*, where adult females deposit eggs; similar in size, shape, and color to *S. montela* eggs) at the other. According to the literature, *P. chinensis* eggs are not parasitized by *Ooencyrtus* spp. (Fatouros et al. 2020). In the second setup, we placed five untreated *A. contorta* leaves with similar area sizes at one end and five leaves that had been scratched with a pattern wheel, also with similar area sizes, at the other end. In the third setup, we placed five untreated *A. contorta* leaves at one end and five pattern‐wheel‐scratched leaves treated with larval saliva, all of similar size, at the other end. In the last setup, we placed five pattern‐wheel‐scratched leaves at one end and five pattern‐wheel‐scratched leaves treated with larval saliva, both groups of leaves having similar sizes, at the other end.

We collected *S. montela* eggs from AY, and once the parasitoid wasps emerged, we gathered them for use in experiments. The wasps were introduced into the T‐shaped olfactometer immediately after emergence, making additional feeding unnecessary to maintain behavioral consistency. To standardize VOC emissions, leaf treatments, including saliva treatments, were conducted immediately before each olfactometer test. To ensure adequate sample size without overcrowding, 50–70 individual *Ooencyrtus* spp. parasitoids, which are non‐gregarious, were placed at the start of the central arm of the T‐shaped olfactometer and observed individually for a duration of 30 min to avoid double‐counting. This procedure was repeated 10 times for each of the four experimental setups, and the position of each parasitoid was recorded at the end of its observation period. A “choice” was determined when an *Ooencyrtus* spp. crossed a predefined threshold line located at the midpoint of each arm and remained at the end of the arm for at least 20 s. For the purposes of statistical analysis, only the insects that made a choice toward one arm within the first 30 min of observation were included. To avoid bias from possible chemical marking of the tracks, the glass tube was changed after each test. The position of the T‐shaped was rotated 180° after each test to counter any directional bias. The T tubes were cleansed in hot water (70°C) and dried in an oven at 60°C before each experiment. All experiments were conducted under controlled laboratory conditions at 25°C ± 2°C, 50% humidity, and 500 lx under artificial light to minimize the influence of environmental variables, with white paperboard surrounding the olfactometer to prevent visual perturbations and ensure a homogeneous light environment.

### 2.3 GC–MS Analysis

In August 2022, we collected two leaves from each of the total 30 pots of *A. contorta* , with the plant heights about 150 cm (predominant oviposition site). A total of 30 *A. contorta* plants were used for chemical assays, divided into three treatment groups: untreated, mechanically damaged, and larval saliva‐treated leaves, with 10 replicates per group. Leaves from 10 of these pots were scratched with a pattern wheel, another 10 had leaves both scratched with the pattern wheel and subsequently applied with larval saliva, while the leaves from the remaining 10 pots were left untreated. To distinguish VOCs originating from the saliva itself, we analyzed the VOC profile of the saliva separately before applying it to the plant leaves. This analysis identified compounds specific to the saliva, such as 1‐butanol, 3‐methyl‐, benzyl alcohol, caryophyllene, humulene, and neophytadiene. The two leaves were placed in clean individual 20 mL glass vials, and empty vials without any leaves were prepared in the same manner as the leaf samples as controls. Leaf headspace volatiles were collected using polydimethylsiloxane (PDMS) tubing as previously described (Kallenbach et al. 2014). This PDMS tubing exhibits a high affinity for a wide range of volatile compounds, allowing efficient trapping of leaf volatiles from the headspace. Clean strips of PDMS tubing, 1 cm in length, were placed directly inside the vials (including control vials that sampled the surrounding air) and left undisturbed for 24 h. Gas chromatography–mass spectrometry (GC–MS, QP2020, Shimadzu, Kyoto, Japan) connected to a TD‐30 thermal desorption unit (Shimadzu, Kyoto, Japan) was used for volatile analysis. The volatile compounds were separated using an Rtx‐5MS column (30 m x 0.25 mm ID, 0.25 μm film thickness; Shimadzu, Kyoto, Japan). The sample was injected in split mode with a 40:1 split ratio, and helium was used as the carrier gas with a constant linear velocity of 52.9 cm/s. The gas chromatograph oven temperature program started at 40°C for 5 min, then increased to 195°C at a rate of 3°C/min, followed by a further increase to 280°C at a rate of 30°C/min, and held at 280°C for 0.83 min. The mass spectrometer operated in electron ionization (EI) mode, and the mass spectra were acquired in scan mode from m/z 33 to 400. The identification of the compounds was confirmed by matching their mass spectra against the NIST 14 mass spectral library. The quantification of the compounds was performed by integrating the peak areas and normalizing them against the leaf area (cm^2^). The percentage composition of each VOC was calculated by dividing the peak area of each compound by the total peak area of all detected compounds and multiplying the result by 100.

### 2.4 Data Analysis

We used Fisher's exact test to analyze whether differences in parasitism rates among regions were significant. Prior to conducting parametric tests, we assessed the normality of all datasets, including the behavioral assay and VOC emission data, using the Shapiro–Wilk test. We employed analysis of variance (ANOVA) after the homogeneity of variance test and post hoc tests (Duncan's test) to determine the significance of the number of *Ooencyrtus* spp. among the conditions and to evaluate the relative abundance of VOC emissions from *A. contorta* leaves under three treatment conditions, with the significance level set at *p* < 0.05. All observations were collected independently to satisfy the assumption of independence. Pearson's correlation coefficient was employed to evaluate the relationship between the number of *Ooencyrtus* spp. and the number of *S. montela* larvae and eggs, with the significance level set at *p* < 0.01. For the olfactometer bioassay, independent t‐tests were used to analyze differences in the number of *Ooencyrtus* spp. choices between conditions, with significance set at *p* < 0.05. An analysis of variance (ANOVA) was performed to assess the relative abundance of VOC emissions from *A. contorta* leaves under three treatment conditions: control, mechanical damage, and damage with saliva. We utilized SPSS software version 23.0 (SPSS Inc., Chicago, IL, USA) for the statistical analysis.

## 3 Results

### 3.1 Field Survey

**FIGURE 2:**
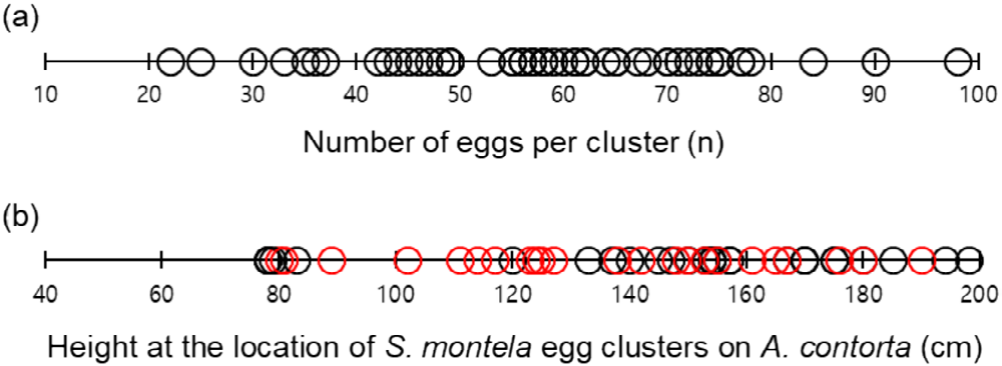
Number of eggs and distribution of *S*. *montela* egg clusters on *A. contorta* : (a) Number of eggs per cluster and (b) height at the location of *S. montela* egg clusters on *A. contorta* (the red circles are parasitized egg clusters).

Our field surveys conducted across the regions revealed significant insights into the oviposition behavior of *S. montela* on *A. contorta* . *S. montela* laid about 58.7 eggs at once (in a single cluster) (Figure 2a). Egg clusters were commonly found at heights between 10 to 200 cm above ground level, with a majority located at approximately 150 cm, indicating a possible preferential height range for oviposition by *S. montela*. The distribution of parasitized clusters is dispersed without preference for the height of *A. contorta* (Figure 2b).

**FIGURE 3:**
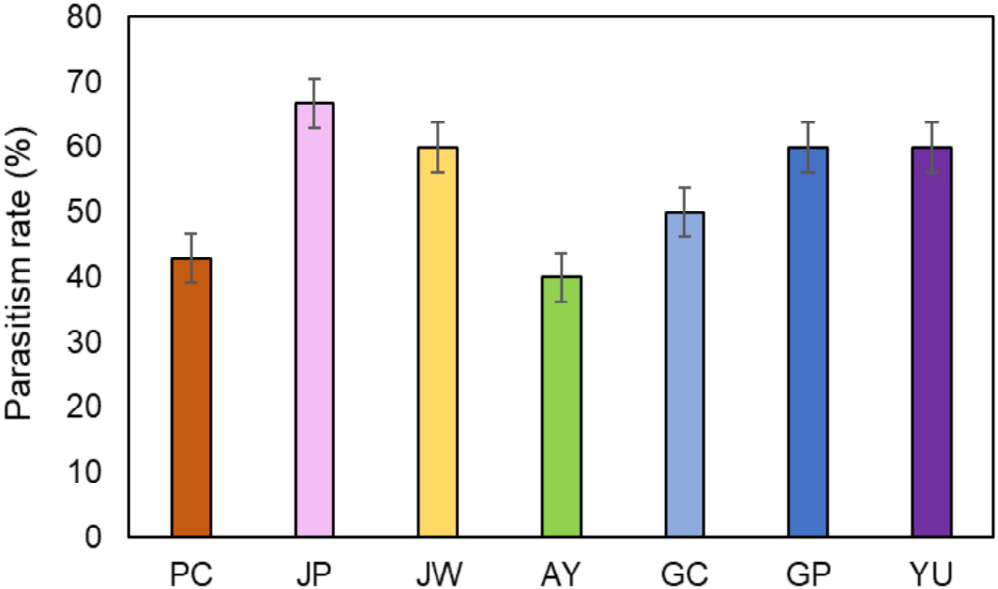
Parasitism rate (%) of *S. montela* eggs by *Ooencyrtus* spp. by region.

Parasitism rates varied by region, with a noteworthy presence of *Ooencyrtus* spp. parasites identified (Figure 3). The overall parasitism rate observed across all regions is around 54.6%. Among the regions, JP exhibits the highest parasitism rate at 66.7%, followed by GP and YU both at 60.0%, JW at 60.0%, GC at 50.0%, PC at 42.9%, and finally, AY with the lowest rate at 40.0%. Regional differences in parasitism rates were not statistically significant (*p* = 0.935).

**FIGURE 4:**
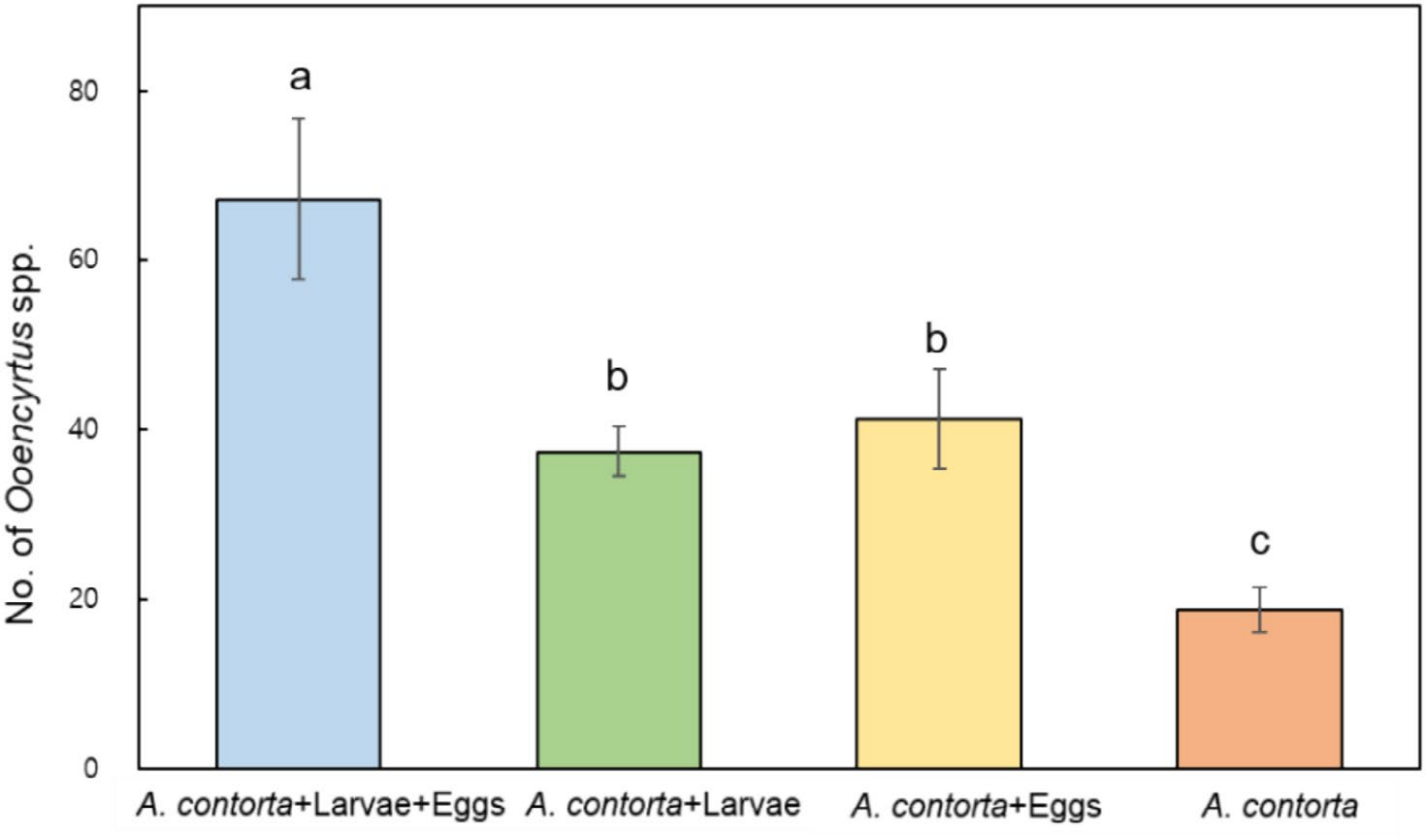
Sticky trap captures of *Ooencyrtus* spp. across different conditions. Letters on the graph indicate significant differences at the 5% level, based on Duncan's test. Bars indicate standard errors.

The sticky traps installed within the quadrats captured an average of 35 *Ooencyrtus* spp. individuals per trap. The average counts of *Ooencyrtus* spp. individuals were as follows: 67.2 for *A. contorta* with both larvae and eggs, 41.3 for *A. contorta* with eggs only, 37.4 for *A. contorta* with larvae only, and 18.8 for *A. contorta* alone (Figure 4). Using Pearson's correlation, significant correlations were observed between the number of *Ooencyrtus* spp. and *S. montela* larvae (*r* = 0.691) and eggs (*r* = 0.549), both with *p* < 0.01.

### 3.2 Olfactometer Bioassay

**FIGURE 5:**
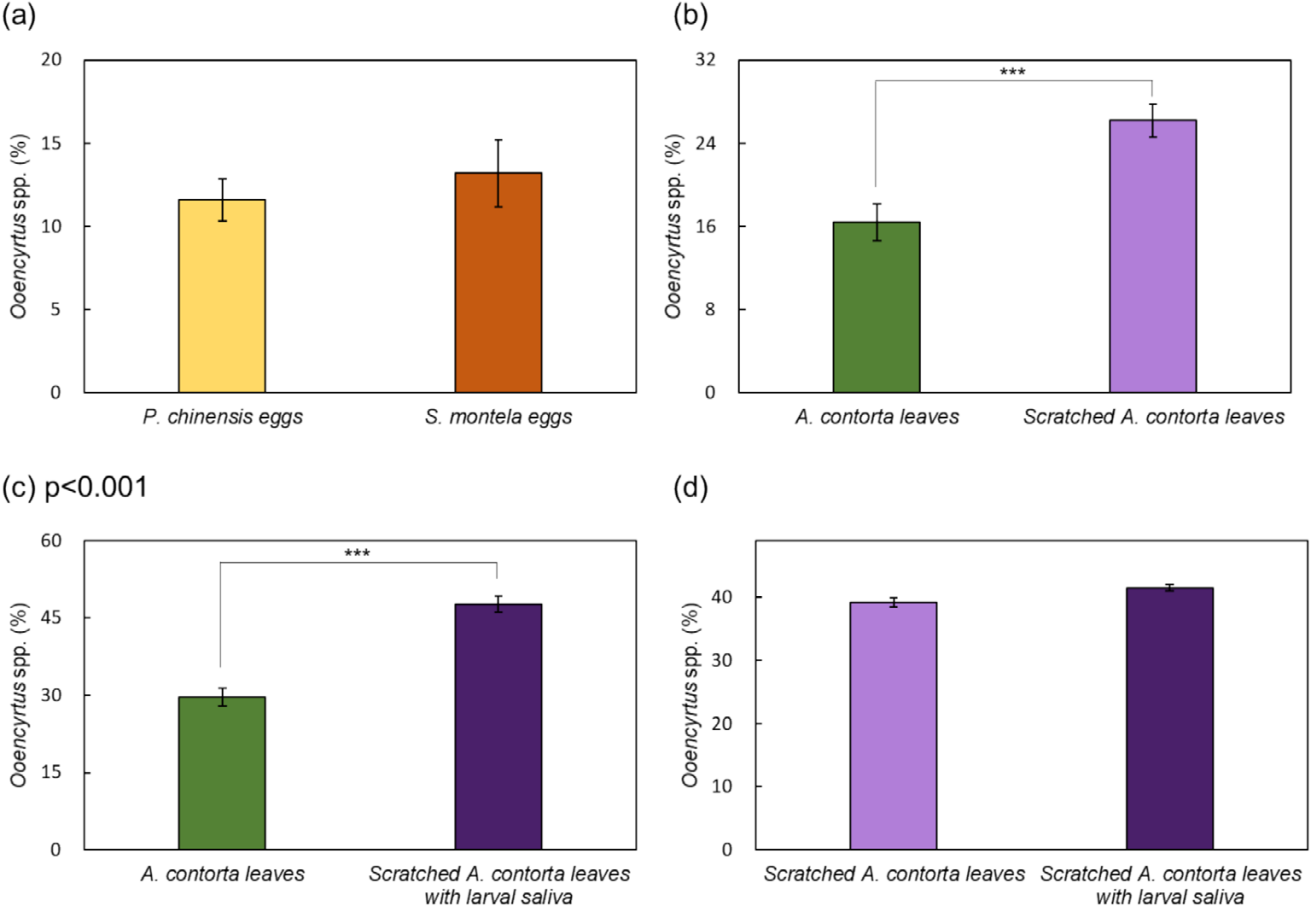
Olfactometer test results revealed the distribution (%) of *Ooencyrtus* spp. choices: (a) Between *S. montela* and *P. chinensis* egg clusters, (b) between control and scratched *A. contorta* leaves, (c) between control and *A. contorta* leaves scratched and treated with larval saliva, and (d) between scratched *A. contorta* leaves and leaves scratched and treated with larval saliva. ***p < 0.001.

When presented with a choice between non‐parasitized *S. montela* egg clusters and *P. chinensis* egg clusters in a T‐shaped olfactometer, *Ooencyrtus* spp. exhibited no significant preference for *S. montela* eggs. Notably, *Ooencyrtus* spp. moved toward *P. chinensis* eggs (11.6%) and *S. montela* eggs (13.2%), with most remaining in the middle section (Figure 5a). In separate trials, *Ooencyrtus* spp. selected *A. contorta* leaves that had been mechanically damaged with a pattern wheel in 46.8% of cases (Figure 5b). When the leaves were both scratched with the pattern wheel and treated with larval saliva, *Ooencyrtus* spp. were found on these leaves in 47.6% of cases (Figure 5c).

### 3.3 VOCs Analysis

**FIGURE 6:**
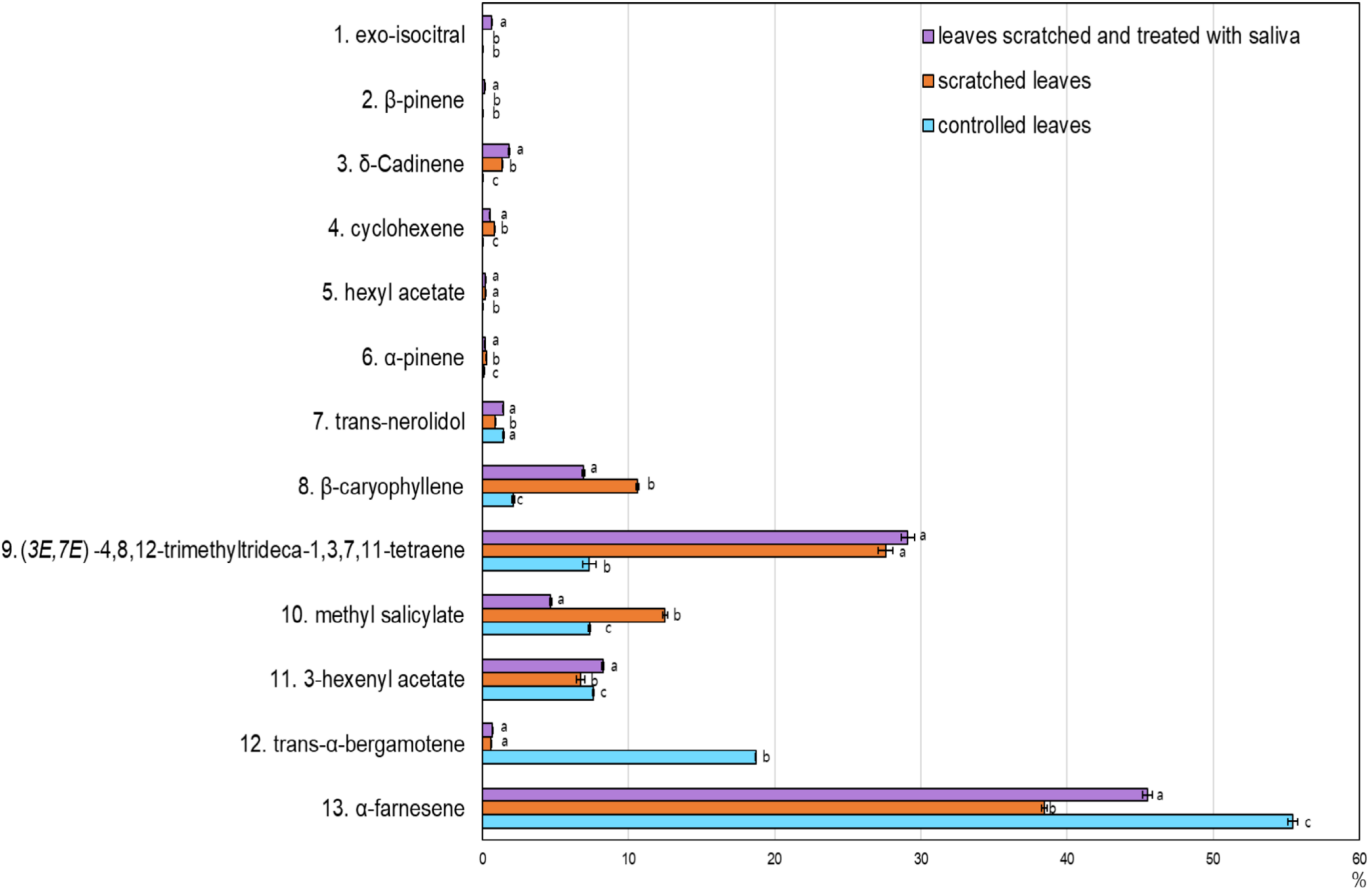
Relative abundance of VOC emissions from *A. contorta* leaves: Controlled, mechanically damaged, and damage with saliva treatment. Letters on the graph indicate significant differences at the 5% level, based on Duncan's test. Bars indicate standard errors.

VOCs analysis of *A. contorta* leaves revealed distinct profiles between the controlled leaves, scratched leaves, and scratched leaves with larval saliva (Table 1, Figure 6). VOCs analysis of *A. contorta* leaves revealed distinct profiles among controlled leaves, scratched leaves, and scratched leaves with larval saliva. exo‐isocitral was present at 0.61% after saliva treatment but was not detected in controlled or scratched leaves. β‐pinene appeared at 0.14% after saliva treatment, although it was absent in both controlled and scratched leaves. δ‐cadinene was detected at 1.36% after scratching and increased to 1.81% with saliva application. Cyclohexene was present at 0.84% following scratching but decreased to 0.52% with saliva treatment. Hexyl acetate was recorded at 0.21% after scratching and decreased slightly to 0.20% with saliva. α‐pinene concentration rose by 0.19% due to scratching and then decreased by 0.10% with saliva treatment, while β‐caryophyllene increased by 8.49% after scratching and decreased by 3.69% after saliva was applied. the compound (3*E*,7*E*)‐4,8,12‐trimethyltrideca‐1,3,7,11‐tetraene showed an increase of 20.28% after scratching and a further increase of 1.51% with saliva treatment. methyl salicylate rose by 5.17% with scratching but decreased to 4.68% following saliva application. 3‐hexenyl acetate decreased by 0.87% with scratching but rose significantly to 8.24% with saliva treatment. trans‐α‐bergamotene dropped by 18.10% post‐scratching, followed by a slight increase of 0.10% with saliva. Lastly, α‐farnesene decreased by 17.01% after scratching and increased by 7.07% after treatment with saliva.

**TABLE 1:** Peak area and chemical composition of the VOCs in different treatments of *A. contorta* (RT, retention time (m)).

Peaks	RT (m)	Compounds	peak area/cm^2^ (percentage composition %)					
			Controlled (%)		Scratched (%)		Scratched and saliva‐treated (%)	
1	9.725	α‐pinene	64,641	(0.08)	234,870	(0.27)	135,085	(0.17)
2	11.865	β‐pinene	—	—	—	—	113,554	(0.14)
3	13.825	3‐hexen‐1‐ol, acetate, (E)—	6,018,406	(7.57)	5,790,356	(6.70)	6,584,443	(8.24)
4	14.23	hexyl acetate	—	—	177,590	(0.21)	158,275	(0.20)
5	19.475	6‐octenal, 7‐methyl‐3‐methylene—	—	—	—	—	489,351	(0.61)
6	23.2	methyl salicylate	5,830,349	(7.33)	10,799,302	(12.50)	3,741,856	(4.68)
7	33.58	caryophyllene	1,683,355	(2.12)	9,164,698	(10.61)	5,528,845	(6.91)
8	35.05	cyclohexene	—	—	722,251	(0.84)	412,447	(0.52)
9	37.53	α‐farnesene	44,090,633	(55.45)	33,207,239	(38.44)	36,388,513	(45.51)
10	37.575	trans‐α‐bergamotene	14,871,422	(18.70)	516,767	(0.60)	556,475	(0.70)
11	38.095	naphthalene, 1,2,3,5,6,8a‐hexahydro‐4,7‐dimethyl‐1‐(1‐methylethyl)‐, (1 s‐cis)—	—	—	1,178,192	(1.36)	1,443,774	(1.81)
12	39.745	1,6,10‐dodecatrien‐3‐ol, 3,7,11‐trimethyl‐, (E)—	1,149,828	(1.45)	761,566	(0.88)	1,133,102	(1.42)
13	40.382	(3*E*,7*E*)‐4,8,12‐trimethyltrideca‐1,3,7,11‐tetraene	5,808,498	(7.30)	23,833,582	(27.59)	23,269,958	(29.10)

## 4 Discussion

Our study found that *S. montela* lays eggs at specific heights on *A. contorta* , with a consistently high parasitism rate across regions. Sticky traps captured more *Ooencyrtus* spp. near *A. contorta* with eggs and larvae. Olfactometer tests indicated that *Ooencyrtus* spp. were attracted to mechanically damaged *A. contorta* leaves, but the addition of larval saliva did not significantly enhance their attraction to these leaves. VOC analysis further revealed unique compound changes in response to damage and saliva treatment, suggesting that *A. contorta* 's VOCs play a role in attracting parasitoids.

### 4.1 Field Surveys

The results from our field survey provided a comprehensive overview of the oviposition behavior of *S. montela* on *A. contorta* , revealing that *S. montela* laid an average of 58.7 eggs per cluster, predominantly at heights of 150 cm. This specific height preference for oviposition might be influenced by microclimatic conditions (Braem and Van Dyck 2023), predation pressure (Obermaier et al. 2006), or the physiological state of *A. contorta* at this height, which could offer optimal conditions for the survival of *S. montela*'s offspring (Pocius et al. 2022). The dispersed distribution of parasitized egg clusters suggests that *Ooencyrtus* spp., the parasitoid, did not exhibit a height preference in its parasitism behavior, which could imply a broad searching efficiency across different heights of *A. contorta* .

The variability in parasitism rates across regions, with a general observation of 54.6% and as high as 66.7% in some regions, underscored the significant role of *Ooencyrtus* spp. in regulating *S. montela* populations. These high parasitism rates suggest that *Ooencyrtus* spp. plays a significant role in the natural regulation of *S. montela*, providing valuable insights into the interactions within this ecosystem. Understanding these dynamics can help inform conservation efforts for *S. montela*, while also considering the ecological role of its natural parasitoid, *Ooencyrtus* spp., in regulating its population.

The data from sticky traps installed within the quadrats provided insights into the attraction dynamics of *Ooencyrtus* spp. toward *A. contorta* when associated with *S. montela*. This setup also offered indirect evidence for the role of VOCs. Significantly higher numbers of *Ooencyrtus* spp. were observed in plots containing both *A. contorta* and *S. montela* larvae, suggesting that the VOCs emitted from the combined effects of feeding damage and larval saliva play a crucial role in attracting parasitoids (Davidson‐Lowe and Ali 2021; Gebreziher 2020; Moujahed et al. 2014). The significant increase in the number of *Ooencyrtus* spp. individuals in the presence of *S. montela* larvae or eggs, compared to the absence of *S. montela*, suggests a compound effect where the combined cues from both plants and larvae create a stronger attractant signal for *Ooencyrtus* spp. This is further supported by the strong correlations between *Ooencyrtus* spp. numbers and the presence of *S. montela* larvae (*r* = 0.691, *p* < 0.01) and eggs (*r* = 0.549, *p* < 0.01).

The increase in *Ooencyrtus* spp. in the presence of larvae may be attributed to the VOCs released from leaf damage caused by the feeding larvae. Additionally, as *S. montela* larvae develop from eggs, their presence may indicate areas with high oviposition activity, indirectly influencing *Ooencyrtus* spp. distribution. This suggests that parasitoids may be responding not only to plant volatiles but also to cues associated with high host egg densities. Such findings align with the hypothesis that herbivory‐induced VOCs serve as an essential signal to recruit parasitoids like *Ooencyrtus* spp., supporting the natural regulation of herbivores in this ecological community (Pérez‐Hedo et al. 2021; War et al. 2018). Additionally, the higher numbers associated with eggs may stem from both the emergence of new *Ooencyrtus* spp. and potential oviposition‐induced plant volatiles (OIPVs), which serve as airborne cues attracting parasitoids to oviposition sites, distinct from contact kairomones that require physical interaction for detection (Fatouros et al. 2020; Ganjisaffar and Perring 2020; Reddy and Guerrero 2004). Notably, in our olfactometer results, *S. montela* eggs alone did not attract *Ooencyrtus* spp. more than *P. chinensis* eggs. Therefore, it was suggested that the volatiles emitted by *A. contorta* , particularly after herbivore damage, might signal *Ooencyrtus* spp., enhancing their role in the natural ecological processes involving *S. montela* (Colazza et al. 2022). This interaction illustrated the complex ecological relationships within the ecosystem and exemplified how plant volatiles act as indirect defenses by recruiting the natural enemies of herbivores (Pearse et al. 2020).

### 4.2 Olfactometer Bioassay

Our experiments revealed that *Ooencyrtus* spp. displayed no significant preference between non‐parasitized *S. montela* egg clusters and *P. chinensis* egg clusters, suggesting a nuanced approach to host selection that may rely on more specific or additional cues than those tested. This observation aligned with previous research indicating that parasitoids are adept at navigating complex environments to locate their hosts, often relying on a combination of stimuli, including host chemical signals and plant volatiles from damage, to make definitive host selection decisions and enhance foraging (Bertoldi et al. 2019; Chesnais et al. 2015; Dicke and Baldwin 2010; Koutsogeorgiou et al. 2023).

*Ooencyrtus* spp. significantly preferred mechanically damaged *A. contorta* leaves to undamaged ones, highlighting the importance of DIPVs in parasitoid attraction (Davidson‐Lowe and Ali 2021; Fatouros et al. 2020). Additionally, the introduction of larval saliva to the damaged leaves did not notably alter their attractiveness; *Ooencyrtus* spp. were drawn to mechanically damaged leaves with and without larval saliva treatment in similar proportions (46.8% and 47.6%, respectively). These results suggest that DIPVs provide a more stable and consistent cue for parasitoid recruitment, while herbivore saliva does not significantly contribute to their attraction (Heil 2004; Turlings and Tumlinson 1992).

This contrasts with previous studies where HIPVs were identified as key signals for parasitoid attraction (Takabayashi 2022; Turlings and Tumlinson 1992). VOC analysis revealed that saliva‐treated leaves exhibited some unique compounds (e.g., exo‐isocitral, β‐pinene); however, these did not significantly influence parasitoid behavior. This aligns with findings from other systems, where DIPVs often serve as reliable signals for generalist parasitoids (Mumm and Dicke 2010; Ponzio et al. 2016).

*Ooencyrtus* spp. responds to DIPVs in host location, which may be an adaptive strategy given that its hosts do not produce strong HIPVs (Heil 2004; Kaplan 2012). This adaptation may stem from the ability of specialized herbivores like *S. montela* to suppress HIPV emissions. In response, parasitoids such as *Ooencyrtus* spp. may have evolved to rely more on DIPVs, which provide a more stable and reliable cue for host location (Fatouros et al. 2020; Pearse et al. 2020).

While larval parasitoids such as *Cotesia* and *Campoletis* spp. primarily use HIPVs to locate their hosts (Dicke and Baldwin 2010; Takabayashi and Dicke 1996), *Ooencyrtus* spp. exhibits a different strategy by prioritizing DIPVs. This suggests that its foraging behavior is better suited to environments where herbivore‐derived cues are weak or inconsistent. As an egg parasitoid, *Ooencyrtus* spp. benefits from detecting recent herbivory or oviposition sites, enhancing its foraging efficiency in dynamic environments (Kaplan 2012; Mumm and Dicke 2010). This adaptation underscores the distinct foraging strategy of egg parasitoids, which rely on stable plant volatiles rather than herbivore‐derived cues for efficient host location (Kaplan 2012; Takabayashi and Dicke 1996).

### 4.3 The Role of VOCs in Plant Defense Strategies

The VOCs analysis of *A. contorta* leaves has demonstrated a multifaceted response to physical damage and simulated herbivore activity, showcasing the plant's layered defense mechanisms (Brosset and Blande 2022). Mechanical damage triggered the primary emission of VOCs essential for attracting parasitoids, while saliva‐induced compounds caused minor modifications to the VOC profile, potentially fine‐tuning the defense response (Davidson‐Lowe and Ali 2021; Kaplan 2012). This aligns with the general plant defense mechanism, where calcium ions and plant hormones activate signaling pathways, inducing the production of VOCs that deter herbivores and recruit natural enemies (Lecourieux et al. 2006; Zhang et al. 2014).

Our findings indicate that *A. contorta* primarily relies on DIPVs rather than HIPVs for parasitoid recruitment (Heil 2004; Kaplan 2012). Given that *S. montela* effectively neutralizes *A. contorta* 's direct chemical defenses (Jeong et al. 2023; Palma‐Onetto et al. 2023), this suggests that plants facing specialist herbivores may shift toward indirect defenses based on more stable and generalizable cues such as damage‐induced volatiles (Fatouros et al. 2020; Pearse et al. 2020). This challenges conventional tritrophic interaction models, which typically emphasize HIPVs as primary signals for parasitoid attraction (Dicke and Baldwin 2010). By demonstrating that mechanical damage alone is sufficient to attract parasitoids, our study expands current understanding of how plants adapt their signaling strategies when their direct chemical defenses become ineffective against highly specialized herbivores.

Several key VOCs exhibited distinct patterns in response to herbivory. Exo‐isocitral was absent in control and mechanically damaged leaves but emerged following saliva treatment, suggesting a saliva‐induced biosynthetic pathway (de Souza Alves et al. 2019). β‐pinene and δ‐cadinene increased with mechanical damage, whereas cyclohexene decreased upon saliva application, highlighting a targeted response to herbivory (Degenhardt 2009; Shen et al. 2000; Shi et al. 2022). Additionally, hexyl acetate, which appeared post‐damage, may serve as an attractant for beneficial insects, while α‐pinene exhibited a rapid response to damage, subsequently modulated by saliva (Chan et al. 2016; Mofikoya et al. 2019; Wang et al. 2013).

Several key VOCs exhibited distinct patterns in response to herbivory. β‐pinene was absent in control and mechanically damaged leaves but appeared following saliva treatment, indicating a potential role in herbivore‐induced signaling (de Souza Alves et al. 2019). Similarly, δ‐cadinene, which is associated with plant defense responses, increased following mechanical damage but showed a slight decrease upon saliva application, suggesting a nuanced role in plant defense modulation (Li et al. 2022; Shen et al. 2000; Yang et al. 2011). Additionally, (3*E*,7*E*)‐4,8,12‐trimethyltrideca‐1,3,7,11‐tetraene was significantly elevated following damage and saliva application, suggesting a role in intra‐plant signaling and herbivore deterrence signaling (Schröder et al. 2015; Shi et al. 2022).

Methyl salicylate, known for its dual function in attracting parasitoids and providing antifungal properties, was found in higher concentrations post‐damage, reflecting the versatility of *A. contorta* 's VOC‐mediated defenses (Tang et al. 2015). 3‐hexenyl acetate, a compound commonly associated with herbivore‐induced signaling, increased upon saliva application, indicating a response to active herbivory. Further analysis revealed that trans‐nerolidol, trans‐α‐bergamotene, and α‐farnesene were differentially regulated following mechanical damage and saliva treatment. These compounds are widely recognized for their roles in attracting predatory insects and parasitoids (Krips 2000; Ozawa et al. 2013; Xiao et al. 2023).

Notably, β‐caryophyllene, which plays a key role in plant defense by acting as a foraging cue for parasitoids, exhibited significant variation, reinforcing its role in indirect plant defenses (Chan et al. 2016; Degenhardt 2009; Xiao et al. 2012). Hexyl acetate, appearing post‐damage, may serve as an attractant for beneficial insects, while α‐pinene exhibited a rapid response to damage, subsequently modulated by saliva (Chan et al. 2016; Mofikoya et al. 2020; Wang et al. 2013).

Despite these dynamic VOC responses, saliva application did not significantly enhance the attraction of *Ooencyrtus* spp., indicating that mechanical damage alone provides sufficient cues for parasitoid recruitment (Colazza et al. 2022; Kaplan 2012). This finding is further supported by our separate VOC analysis of *S. montela* saliva, which revealed distinct compounds—1‐butanol, 3‐methyl‐, benzyl alcohol, caryophyllene, humulene, and neophytadiene—that were absent in the VOC profile of treated leaves. This suggests that the observed differences in VOC emissions originated primarily from plant responses to damage rather than direct contributions from herbivore saliva.

These results emphasize that plants experiencing pressure from specialist herbivores may evolve alternative defense strategies, prioritizing mechanically induced signals over HIPVs to ensure reliable parasitoid attraction (Gish et al. 2015; Mofikoya et al. 2020). Understanding the specific functions of VOCs in plant‐herbivore interactions is critical for ecological resilience and pest management applications, providing insights into how plants modulate their defense strategies in response to evolutionary pressures (Dicke and Baldwin 2010; Fatouros et al. 2020).

### 4.4 Ecological and Evolutionary Implications

Our findings support the broader framework of the evolutionary arms race between plants, herbivores, and their natural enemies (Endara et al. 2023; Kobayashi et al. 2015; Pearse et al. 2020). *A. contorta* appears to have evolved an indirect defense strategy that prioritizes DIPVs over OIPVs, possibly as a counteradaptation to specialist herbivores that suppress HIPV production (Fatouros et al. 2020; Heil 2004). This adaptation aligns with the escape‐and‐radiate coevolution model, in which plants develop new defense mechanisms in response to herbivore adaptations, subsequently influencing parasitoid foraging strategies (Futuyma and Agrawal 2009; Stam et al. 2014; Thompson et al. 2022). By favoring DIPVs over herbivore‐associated cues, *A. contorta* may increase the reliability of its indirect defenses, ensuring that parasitoids such as *Ooencyrtus* spp. are recruited effectively even when herbivores manipulate or suppress volatile emissions (Kaplan 2012; Mumm and Dicke 2010).

Specifically, the VOCs emitted in response to *S. montela* larval feeding played a significant role in attracting parasitoids such as *Ooencyrtus* spp., which specialize in locating herbivores in active feeding stages (War et al. 2012). For an organism like *Ooencyrtus* spp., which parasitizes eggs, the absence of strong oviposition cues suggested an adaptive reliance on feeding‐associated VOCs, allowing it to identify hosts with a high likelihood of freshly laid eggs (Gebreziher 2018; Heil 2004).

Timing was crucial in this interaction. Since *Ooencyrtus* spp. needed to locate eggs shortly after they were laid to maximize reproductive success, aligning its host‐seeking behavior with *S. montela*'s life cycle was essential (Degenhardt 2009; Heil 2004). Larval feeding VOCs emitted by *A. contorta* served as indicators of the recent presence of eggs, providing a temporal cue that potentially increased the accuracy of *Ooencyrtus* spp. in locating viable eggs (Gebreziher 2018). This reliance on larval cues over direct oviposition cues reflected a highly specialized adaptation: rather than responding to every oviposition event, *Ooencyrtus* spp. may focus on VOCs that indicate a more active infestation, thereby increasing its efficiency and reproductive success (Heil 2004; Pearse et al. 2020).

These chemical signals may serve dual roles by deterring attackers and potentially summoning parasitoids like *Ooencyrtus* spp. to manage herbivore populations such as *S. montela*, though future studies should directly test these behavioral responses to validate their functional impact. This mutual adaptation among *A. contorta, S. montela*, and *Ooencyrtus* spp. demonstrated the intricate role of timing and specificity in chemical ecology. By focusing on herbivore feeding cues, *A. contorta* not only engaged parasitoids more effectively but also promoted a resilient ecosystem, facilitating ecological balance through adaptive chemical communication. This interaction underscored how VOC‐mediated defenses contributed to biodiversity and ecosystem stability, reflecting the evolutionary sophistication of plant‐insect‐parasitoid dynamics (Heil 2004; Krips 2000; Pearse et al. 2020).

## 5 Conclusion

This study revealed the intricate relationship between *A. contorta* , the herbivore *S. montela*, and parasitoids, mediated by a complex VOC signaling system. Our findings indicated that although larval saliva‐induced compounds were released, they did not significantly enhance *A. contorta* 's attractiveness to *Ooencyrtus* spp., suggesting that these specific VOCs may not have played a central role in altering parasitoid behavior. By analyzing VOCs like hexyl acetate, cyclohexene, δ‐cadinene, α‐pinene, and β‐caryophyllene, we uncovered a nuanced signaling network through which *A. contorta* can potentially deter herbivores and engage ecological allies.

Our findings challenge the assumption that herbivore saliva universally enhances parasitoid attraction and suggest that parasitoids may favor more stable, damage‐induced plant volatiles over herbivore‐associated cues in certain ecological contexts. Future research should explore whether this pattern holds across different plant‐herbivore systems and whether parasitoids adjust their foraging strategies in response to herbivore suppression of HIPV emissions. Additionally, experimental manipulation of VOC blends could clarify which specific compounds drive parasitoid recruitment.

## Author Contributions

**Si‐Hyun Park:** conceptualization (lead), data curation (lead), formal analysis (lead), funding acquisition (equal), investigation (lead), methodology (lead), resources (lead), software (lead), writing – original draft (lead). **Jae Yeon Jang:** investigation (supporting), methodology (supporting), resources (supporting). **Hangah Lim:** data curation (supporting), formal analysis (supporting), investigation (supporting), methodology (supporting), software (supporting), writing – review and editing (supporting). **Sang‐Gyu Kim:** data curation (supporting), investigation (supporting), methodology (supporting), writing – review and editing (supporting). **Jae Geun Kim:** conceptualization (equal), funding acquisition (lead), project administration (lead), writing – review and editing (equal).

## Conflicts of Interest

The authors declare no conflicts of interest.

## Data Availability

The article contains all the data that were created and used in this investigation, and the corresponding authors can provide these data upon request.

## References

[ece371175-bib-0001] Bashir, H. , A. Ammar , S. Bashir , A. Hassan , and M. Rashid . 2023. “Insects as Allies: The Role of Beneficial Insects in Sustainable Agriculture.” Trends in Animal and Plant Sciences 23, no. 2: 17–24.

[ece371175-bib-0002] Bertoldi, V. , G. Rondoni , J. Brodeur , and E. Conti . 2019. “An Egg Parasitoid Efficiently Exploits Cues From a Coevolved Host but Not Those From a Novel Host.” Frontiers in Physiology 10: 443145. 10.3389/fphys.2019.00746 PMC6621923 31333475

[ece371175-bib-0003] Braem, S. , and H. Van Dyck . 2023. “Larval and Adult Experience and Ecotype Affect Oviposition Behavior in a Niche‐Expanding Butterfly.” Behavioral Ecology 34, no. 4: arad022. 10.1093/beheco/arad022.

[ece371175-bib-0004] Brosset, A. , and J. D. Blande . 2022. “Volatile‐Mediated Plant–Plant Interactions: Volatile Organic Compounds as Modulators of Receiver Plant Defence, Growth, and Reproduction.” Journal of Experimental Botany 73, no. 2: 511–528. 34791168 10.1093/jxb/erab487 PMC8757495

[ece371175-bib-0005] Chan, W.‐K. , L. T.‐H. Tan , K.‐G. Chan , L.‐H. Lee , and B.‐H. Goh . 2016. “Nerolidol: A Sesquiterpene Alcohol With Multi‐Faceted Pharmacological and Biological Activities.” Molecules 21, no. 5: 529. 10.3390/molecules21050529. 27136520 PMC6272852

[ece371175-bib-0006] Chesnais, Q. , A. Ameline , G. Doury , V. Le Roux , and A. Couty . 2015. “Aphid Parasitoid Mothers Don't Always Know Best Through the Whole Host Selection Process.” PLoS One 10, no. 8: e0135661. 26270046 10.1371/journal.pone.0135661 PMC4535949

[ece371175-bib-0007] Colazza, S. , M. L. Pappas , A. M. Cortesero , and C. Rodriguez‐Saona . 2022. “Chemical Ecology and Conservation Biological Control.” Frontiers in Ecology and Evolution 10: 857438.

[ece371175-bib-0008] Conti, E. , G. Avila , B. Barratt , et al. 2021. “Biological Control of Invasive Stink Bugs: Review of Global State and Future Prospects.” Entomologia Experimentalis et Applicata 169, no. 1: 28–51. 10.1111/eea.12967.

[ece371175-bib-0009] Davidson‐Lowe, E. , and J. G. Ali . 2021. “Herbivore‐Induced Plant Volatiles Mediate Behavioral Interactions Between a Leaf‐Chewing and a Phloem‐Feeding Herbivore.” Basic and Applied Ecology 53: 39–48.

[ece371175-bib-0010] de Souza Alves, M. , I. M. Campos , D. d. M. C. de Brito , C. M. Cardoso , E. G. Pontes , and M. A. A. de Souza . 2019. “Efficacy of Lemongrass Essential Oil and Citral in Controlling *Callosobruchus Maculatus* (Coleoptera: Chrysomelidae), a Post‐Harvest Cowpea Insect Pest.” Crop Protection 119: 191–196.

[ece371175-bib-0011] Degenhardt, J. r. 2009. “Indirect Defense Responses to Herbivory in Grasses.” Plant Physiology 149, no. 1: 96–102. 19126700 10.1104/pp.108.128975 PMC2613722

[ece371175-bib-0012] Dicke, M. , and I. T. Baldwin . 2010. “The Evolutionary Context for Herbivore‐Induced Plant Volatiles: Beyond the ‘Cry for Help’.” Trends in Plant Science 15, no. 3: 167–175. 20047849 10.1016/j.tplants.2009.12.002

[ece371175-bib-0013] Endara, M.‐J. , D. L. Forrister , and P. D. Coley . 2023. “The Evolutionary Ecology of Plant Chemical Defenses: From Molecules to Communities.” Annual Review of Ecology, Evolution, and Systematics 54: 107–127.

[ece371175-bib-0014] Fatouros, N. , A. Cusumano , F. Bin , A. Polaszek , and J. Van Lenteren . 2020. “How to Escape From Insect Egg Parasitoids: A Review of Potential Factors Explaining Parasitoid Absence Across the Insecta.” Proceedings of the Royal Society B 287, no. 1931: 20200344. 32693731 10.1098/rspb.2020.0344 PMC7423650

[ece371175-bib-0015] Ferreira, F. V. , and M. A. Musumeci . 2021. “Trichoderma as Biological Control Agent: Scope and Prospects to Improve Efficacy.” World Journal of Microbiology and Biotechnology 37, no. 5: 90. 33899136 10.1007/s11274-021-03058-7

[ece371175-bib-0016] Futuyma, D. J. , and A. A. Agrawal . 2009. “Macroevolution and the Biological Diversity of Plants and Herbivores.” Proceedings of the National Academy of Sciences 106, no. 43: 18054–18061. 10.1073/pnas.0904106106 PMC2775342 19815508

[ece371175-bib-0017] Ganjisaffar, F. , and T. M. Perring . 2020. “Life History Evaluation of Ooencyrtus Lucidus, a Newly Described Egg Parasitoid of Bagrada Hilaris.” Insects 11, no. 5: 292. 32397448 10.3390/insects11050292 PMC7290786

[ece371175-bib-0018] Gardner, S. N. , and A. A. Agrawal . 2002. “Induced Plant Defence and the Evolution of Counter‐Defences in Herbivores.” Evolutionary Ecology Research 4: 1131–1151.

[ece371175-bib-0019] Gebreziher, H. G. 2018. “The Role of Herbivore‐Induced Plant Volatiles (HIPVs) as Indirect Plant Defense Mechanism in a Diverse Plant and Herbivore Species; A Review.” International Journal of Agriculture Environment and Food Sciences 2, no. 4: 139–147.

[ece371175-bib-0020] Gebreziher, H. G. 2020. “Advances in Herbivore‐Induced Plant Volatiles (HIPVs) as Plant Defense and Application Potential for Crop Protection.” International Journal of Botany Studies 5, no. 2: 29–36.

[ece371175-bib-0021] Gish, M. , C. M. De Moraes , and M. C. Mescher . 2015. “Herbivore‐Induced Plant Volatiles in Natural and Agricultural Ecosystems: Open Questions and Future Prospects.” Current Opinion in Insect Science 9: 1–6. 32846702 10.1016/j.cois.2015.04.001

[ece371175-bib-0022] Halitschke, R. , U. Schittko , G. Pohnert , W. Boland , and I. T. Baldwin . 2001. “Molecular Interactions Between the Specialist Herbivore *Manduca Sexta* (Lepidoptera, Sphingidae) and Its Natural Host *Nicotiana Attenuata*. III. Fatty Acid‐Amino Acid Conjugates in Herbivore Oral Secretions Are Necessary and Sufficient for Herbivore‐Specific Plant Responses.” Plant Physiology 125, no. 2: 711–717. 10.1104/pp.125.2.711. 11161028 PMC64872

[ece371175-bib-0023] Heil, M. 2004. “Induction of Two Indirect Defences Benefits Lima Bean ( *Phaseolus lunatus* , Fabaceae) in Nature.” Journal of Ecology 92, no. 3: 527–536.

[ece371175-bib-0024] Hilker, M. , and T. Meiners . 2010. “How Do Plants “Notice” Attack by Herbivorous Arthropods?” Biological Reviews 85, no. 2: 267–280. 19961475 10.1111/j.1469-185X.2009.00100.x

[ece371175-bib-0025] Hu, L. , K. Zhang , Z. Wu , J. Xu , and M. Erb . 2021. “Plant Volatiles as Regulators of Plant Defense and Herbivore Immunity: Molecular Mechanisms and Unanswered Questions.” Current Opinion in Insect Science 44: 82–88. 33894408 10.1016/j.cois.2021.03.010

[ece371175-bib-0026] Jeong, S. J. , B. E. Nam , H. J. Jeong , J. Y. Jang , Y. Joo , and J. G. Kim . 2023. “Age‐Dependent Resistance of a Perennial Herb, Aristolochia Contorta Against Specialist and Generalist Leaf‐Chewing Herbivores.” Frontiers in Plant Science 14: 1145363. 37324666 10.3389/fpls.2023.1145363 PMC10265686

[ece371175-bib-0027] Kallenbach, M. , Y. Oh , E. J. Eilers , D. Veit , I. T. Baldwin , and M. C. Schuman . 2014. “A Robust, Simple, High‐Throughput Technique for Time‐Resolved Plant Volatile Analysis in Field Experiments.” Plant Journal 78, no. 6: 1060–1072. 10.1111/tpj.12523 PMC4190661 24684685

[ece371175-bib-0028] Kaplan, I. 2012. “Trophic Complexity and the Adaptive Value of Damage‐Induced Plant Volatiles.” PLoS Biology 10, no. 11: e1001437. 23209381 10.1371/journal.pbio.1001437 PMC3507926

[ece371175-bib-0029] Kobayashi, C. , K. Matsuo , K. Watanabe , et al. 2015. “Arms Race Between Leaf Rollers and Parasitoids: Diversification of Plant‐Manipulation Behavior and Its Consequences.” Ecological Monographs 85, no. 2: 253–268.

[ece371175-bib-0030] Koutsogeorgiou, E. I. , T. Moysiadis , G. T. Fifis , N. E. Gogolashvili , D. Chatzimpalasis , and S. S. Andreadis . 2023. “Age‐and Density‐Dependent Parasitism Rate and Development Time of the Generalist Egg‐Parasitoid Ooencyrtus Telenomicida (Hymenoptera: Encyrtidae) on Eggs of the Brown Marmorated Stink Bug, *Halyomorpha Halys* .” Insects 15, no. 1: 14. 10.3390/insects15010014. 38249020 PMC10817064

[ece371175-bib-0084] Krips, O. E. 2000. Plant Effects on Biological Control of Spider Mites in the Ornamental Crop Gerbera. Wageningen University and Research.

[ece371175-bib-0032] Lecourieux, D. , R. Ranjeva , and A. Pugin . 2006. “Calcium in Plant Defence‐Signalling Pathways.” New Phytologist 171, no. 2: 249–269. 16866934 10.1111/j.1469-8137.2006.01777.x

[ece371175-bib-0033] Lesieur, V. , and A. O. Farinha . 2021. “Responses of Native Egg Parasitoids to the Invasive Seed Bug *Leptoglossus Occidentalis* .” Agricultural and Forest Entomology 23, no. 3: 323–333. 10.1111/afe.12434.

[ece371175-bib-0034] Li, X. , L. Qi , N. Zang , et al. 2022. “Integrated Metabolome and Transcriptome Analysis of the Regulatory Network of Volatile Ester Formation During Fruit Ripening in Pear.” Plant Physiology and Biochemistry 185: 80–90. 35661588 10.1016/j.plaphy.2022.04.030

[ece371175-bib-0035] Mahawer, S. K. , S. Arya , T. Kabdal , et al. 2022. “Plant Defense Systems: Mechanism of Self‐Protection by Plants Against Pathogens.” Plant Protection: From Chemicals to Biologicals 115: 115–140.

[ece371175-bib-0036] Mathur, V. , P. G. Sinha , and S. A. Noor . 2024. “Unraveling the Coevolutionary Arms Race: Insights Into the Dynamic Interplay of Plants, Insects and Associated Organisms.” In Plant Resistance to Insects in Major Field Crops, 13–36. Springer.

[ece371175-bib-0037] McGale, E. , C. Diezel , M. C. Schuman , and I. T. Baldwin . 2018. “Cry1Ac Production Is Costly for Native Plants Attacked by Non‐Cry1Ac‐Targeted Herbivores in the Field.” New Phytologist 219, no. 2: 714–727. 29754424 10.1111/nph.15207

[ece371175-bib-0038] Mofikoya, A. O. , T. N. T. Bui , M. Kivimäenpää , J. K. Holopainen , S. J. Himanen , and J. D. Blande . 2019. “Foliar Behaviour of Biogenic Semi‐Volatiles: Potential Applications in Sustainable Pest Management.” Arthropod‐Plant Interactions 13, no. 2: 193–212.

[ece371175-bib-0039] Mofikoya, A. O. , P. Yli‐Pirilä , M. Kivimäenpää , J. D. Blande , A. Virtanen , and J. K. Holopainen . 2020. “Deposition of α‐Pinene Oxidation Products on Plant Surfaces Affects Plant VOC Emission and Herbivore Feeding and Oviposition.” Environmental Pollution 263: 114437. 32268226 10.1016/j.envpol.2020.114437

[ece371175-bib-0040] Moujahed, R. , F. Frati , A. Cusumano , et al. 2014. “Egg Parasitoid Attraction Toward Induced Plant Volatiles Is Disrupted by a Non‐Host Herbivore Attacking Above or Belowground Plant Organs.” Frontiers in Plant Science 5: 601. 10.3389/fpls.2014.00601. 25414714 PMC4220641

[ece371175-bib-0041] Mumm, R. , and M. Dicke . 2010. “Variation in Natural Plant Products and the Attraction of Bodyguards Involved in Indirect Plant Defense.” Canadian Journal of Zoology 88, no. 7: 628–667.

[ece371175-bib-0042] Obermaier, E. , A. Heisswolf , B. Randlkofer , and T. Meiners . 2006. “Enemies in Low Places–Insects Avoid Winter Mortality and Egg Parasitism by Modulating Oviposition Height.” Bulletin of Entomological Research 96, no. 4: 337–343. 16923200

[ece371175-bib-0043] Ohnmeiss, T. E. , and I. T. Baldwin . 1994. “The Allometry of Nitrogen to Growth and an Inducible Defense Under Nitrogen‐Limited Growth.” Ecology 75, no. 4: 995–1002.

[ece371175-bib-0044] Ozawa, R. , K. Shiojiri , K. Matsui , and J. Takabayashi . 2013. “Intermittent Exposure to Traces of Green Leaf Volatiles Triggers the Production of (Z)‐3‐Hexen‐1‐Yl Acetate and (Z)‐3‐Hexen‐1‐Ol in Exposed Plants.” Plant Signaling & Behavior 8, no. 11: e27013. 24301200 10.4161/psb.27013 PMC4091332

[ece371175-bib-0085] Palma‐Onetto, V. , J. Bergmann , and M. González‐Teuber . 2023. “Mode of Action, Chemistry and Defensive Efficacy of the Osmeterium in the Caterpillar *Battus polydamas* Archidamas.” Scientific Reports 13, no. 1: 6644. 37095102 10.1038/s41598-023-33390-x PMC10126055

[ece371175-bib-0046] Palma‐Onetto, V. , J. Bergmann , and M. González‐Teuber . 2024. “Author Correction: Mode of Action, Chemistry and Defensive Efficacy of the Osmeterium in the Caterpillar Battus Polydamas Archidamas.” Scientific Reports 14, no. 1: 8791. 10.1038/s41598-024-59540-3. 38627571 PMC11021423

[ece371175-bib-0047] Park, S.‐H. , and J. G. Kim . 2023. “Mechanistic Understanding of Perianth Traits Hindering Pollination in Aristolochia Contorta Bunge.” Frontiers in Plant Science 14: 1226331. 37810400 10.3389/fpls.2023.1226331 PMC10552756

[ece371175-bib-0048] Park, S.‐H. , and J. G. Kim . 2024. “The Reduced Growth due to Elevated CO_2_ Concentration Hinders the Sexual Reproduction of Mature Northern Pipevine (Aristolochia Contorta Bunge).” Frontiers in Plant Science 15: 1359783. 38571710 10.3389/fpls.2024.1359783 PMC10987783

[ece371175-bib-0049] Park, S.‐H. , J. H. Kim , and J. G. Kim . 2023. “Effects of Human Activities on Sericinus Montela and Its Host Plant Aristolochia Contorta.” Scientific Reports 13, no. 1: 8289. 37217596 10.1038/s41598-023-35607-5 PMC10202907

[ece371175-bib-0050] Park, S.‐H. , B. E. Nam , and J. G. Kim . 2019. “Shade and Physical Support Are Necessary for Conserving the Aristolochia Contorta Population.” Ecological Engineering 135: 108–115.

[ece371175-bib-0051] Paudel Timilsena, B. , I. Seidl‐Adams , and J. H. Tumlinson . 2020. “Herbivore‐Specific Plant Volatiles Prime Neighboring Plants for Nonspecific Defense Responses.” Plant, Cell & Environment 43, no. 3: 787–800. 10.1111/pce.13688 31759336

[ece371175-bib-0052] Pearse, I. S. , E. LoPresti , R. N. Schaeffer , et al. 2020. “Generalising Indirect Defence and Resistance of Plants.” Ecology Letters 23, no. 7: 1137–1152. 32394591 10.1111/ele.13512

[ece371175-bib-0053] Pérez‐Hedo, M. , M. Alonso‐Valiente , S. Vacas , et al. 2021. “Plant Exposure to Herbivore‐Induced Plant Volatiles: A Sustainable Approach Through Eliciting Plant Defenses.” Journal of Pest Science 94, no. 4: 1221–1235.

[ece371175-bib-0054] Pocius, V. M. , S. Cibotti , S. Ray , et al. 2022. “Impacts of Larval Host Plant Species on Dispersal Traits and Free‐Flight Energetics of Adult Butterflies.” Communications Biology 5, no. 1: 469. 35577926 10.1038/s42003-022-03396-8 PMC9110344

[ece371175-bib-0055] Poelman, E. H. , A. M. Oduor , C. Broekgaarden , et al. 2009. “Field Parasitism Rates of Caterpillars on *Brassica Oleracea* Plants Are Reliably Predicted by Differential Attraction of Cotesia Parasitoids.” Functional Ecology 23, no. 5: 951–962.

[ece371175-bib-0056] Ponzio, C. , B. T. Weldegergis , M. Dicke , and R. Gols . 2016. “Compatible and Incompatible Pathogen–Plant Interactions Differentially Affect Plant Volatile Emissions and the Attraction of Parasitoid Wasps.” Functional Ecology 30, no. 11: 1779–1789.

[ece371175-bib-0057] Power, N. , F. Ganjisaffar , K. Xu , and T. M. Perring . 2021. “Effects of Parasitoid Age, Host Egg Age, and Host Egg Freezing on Reproductive Success of Ooencyrtus Mirus (Hymenoptera: Encyrtidae) on Bagrada Hilaris (Hemiptera: Pentatomidae) Eggs.” Environmental Entomology 50, no. 1: 58–68. 33219688 10.1093/ee/nvaa150

[ece371175-bib-0058] Reddy, G. V. , and A. Guerrero . 2004. “Interactions of Insect Pheromones and Plant Semiochemicals.” Trends in Plant Science 9, no. 5: 253–261. 15130551 10.1016/j.tplants.2004.03.009

[ece371175-bib-0059] Rondoni, G. , I. Borges , J. Collatz , et al. 2021. “Exotic Ladybirds for Biological Control of Herbivorous Insects–a Review.” Entomologia Experimentalis et Applicata 169, no. 1: 6–27. 10.1111/eea.12963.

[ece371175-bib-0060] Schröder, M. L. , R. Glinwood , B. Webster , R. Ignell , and K. Krüger . 2015. “Olfactory Responses of R Hopalosiphum Padi to Three Maize, Potato, and Wheat Cultivars and the Selection of Prospective Crop Border Plants.” Entomologia Experimentalis et Applicata 157, no. 2: 241–253.

[ece371175-bib-0061] Sehrawat, A. , S. S. Sindhu , and B. R. Glick . 2022. “Hydrogen Cyanide Production by Soil Bacteria: Biological Control of Pests and Promotion of Plant Growth in Sustainable Agriculture.” Pedosphere 32, no. 1: 15–38.

[ece371175-bib-0062] Shen, B. , Z. Zheng , and H. K. Dooner . 2000. “A Maize Sesquiterpene Cyclase Gene Induced by Insect Herbivory and Volicitin: Characterization of Wild‐Type and Mutant Alleles.” Proceedings of the National Academy of Sciences 97, no. 26: 14807–14812. 10.1073/pnas.240284097 PMC19000 11106370

[ece371175-bib-0063] Shi, M.‐Z. , J.‐Y. Li , Y.‐T. Chen , L. Fang , H. Wei , and J.‐W. Fu . 2022. “Plant Volatile Compounds of the Invasive Alligatorweed, *Alternanthera philoxeroides* (Mart.) Griseb, Infested by *Agasicles hygrophila* Selman and Vogt (Coleoptera: Chrysomelidae).” Life 12, no. 8: 1257. 36013435 10.3390/life12081257 PMC9410005

[ece371175-bib-0064] Shivaramu, S. , P. D. K. Jayanthi , V. Kempraj , R. Anjinappa , B. Nandagopal , and A. K. Chakravarty . 2017. “What Signals Do Herbivore‐Induced Plant Volatiles Provide Conspecific Herbivores?” Arthropod‐Plant Interactions 11, no. 6: 815–823. 10.1007/s11829-017-9536-2.

[ece371175-bib-0065] Stam, J. M. , A. Kroes , Y. Li , et al. 2014. “Plant Interactions With Multiple Insect Herbivores: From Community to Genes.” Annual Review of Plant Biology 65, no. 1: 689–713. 10.1146/annurev-arplant-050213-035937 24313843

[ece371175-bib-0066] Takabayashi, J. 2022. “Herbivory‐Induced Plant Volatiles Mediate Multitrophic Relationships in Ecosystems.” Plant and Cell Physiology 63, no. 10: 1344–1355. 35866611 10.1093/pcp/pcac107

[ece371175-bib-0067] Takabayashi, J. , and M. Dicke . 1996. “Plant—Carnivore Mutualism Through Herbivore‐Induced Carnivore Attractants.” Trends in Plant Science 1, no. 4: 109–113.

[ece371175-bib-0068] Tang, F. , Y.‐Y. Fu , and J.‐R. Ye . 2015. “The Effect of Methyl Salicylate on the Induction of Direct and Indirect Plant Defense Mechanisms in Poplar (Populus× Euramericana ‘Nanlin 895’).” Journal of Plant Interactions 10, no. 1: 93–100.

[ece371175-bib-0069] Thompson, M. N. , R. F. Medina , A. M. Helms , and J. S. Bernal . 2022. “Improving Natural Enemy Selection in Biological Control Through Greater Attention to Chemical Ecology and Host‐Associated Differentiation of Target Arthropod Pests.” Insects 13, no. 2: 160. 35206733 10.3390/insects13020160 PMC8877252

[ece371175-bib-0070] Turlings, T. C. , and J. H. Tumlinson . 1992. “Systemic Release of Chemical Signals by Herbivore‐Injured Corn.” Proceedings of the National Academy of Sciences 89, no. 17: 8399–8402. 10.1073/pnas.89.17.8399 PMC49926 11607325

[ece371175-bib-0071] Wang, S. Y. , S. H. Gu , L. Han , Y. Y. Guo , J. J. Zhou , and Y. J. Zhang . 2013. “Specific Involvement of Two Amino Acid Residues in Cis‐Nerolidol Binding to Odorant‐Binding Protein 5 AlinOBP5 in the Alfalfa Plant Bug, A Delphocoris Lineolatus (Goeze).” Insect Molecular Biology 22, no. 2: 172–182. 10.1111/imb.12012. 23294484

[ece371175-bib-0074] War, A. R. , G. K. Taggar , B. Hussain , M. S. Taggar , R. M. Nair , and H. C. Sharma . 2018. “Plant Defence Against Herbivory and Insect Adaptations.” AoB Plants 10, no. 4: ply037.

[ece371175-bib-0072] War, A. R. , M. G. Paulraj , T. Ahmad , et al. 2012. “Mechanisms of Plant Defense Against Insect Herbivores.” Plant Signaling & Behavior 7, no. 10: 1306–1320. 22895106 10.4161/psb.21663 PMC3493419

[ece371175-bib-0086] War, A. R. , and H. C. Sharma . 2014. “Induced Resistance in Plants and Counter‐Adaptation by Insect Pests.” In Short Views on Insect Biochemistry and Molecular Biology, edited by R. Chandrasekar , B. K. Tyagi , Z. Z. Gui and G. R. Reeck , 533–547. International Book Mission.

[ece371175-bib-0075] Wari, D. , T. Aboshi , T. Shinya , and I. Galis . 2022. “Integrated View of Plant Metabolic Defense With Particular Focus on Chewing Herbivores.” Journal of Integrative Plant Biology 64, no. 2: 449–475. 34914192 10.1111/jipb.13204

[ece371175-bib-0076] Whitehill, J. G. , J. Bohlmann , and P. Krokene . 2023. Forest Entomology and Pathology: Volume 1: Entomology, edited by T. D. Schowalter , 169–204. Springer International Publishing.

[ece371175-bib-0077] Wojda, I. , M. Cytryńska , A. Zdybicka‐Barabas , and J. Kordaczuk . 2020. “Insect Defense Proteins and Peptides.” In Vertebrate and Invertebrate Respiratory Proteins, Lipoproteins and Other Body Fluid Proteins, edited by V. R. Preedy , 81–121. Springer. 10.1007/978-3-030-41769-7_4 32189297

[ece371175-bib-0078] Xiao, Y. , Q. Wang , M. Erb , et al. 2012. “Specific Herbivore‐Induced Volatiles Defend Plants and Determine Insect Community Composition in the Field.” Ecology Letters 15, no. 10: 1130–1139. 10.1111/j.1461-0248.2012.01835.x. 22804824

[ece371175-bib-0079] Xiao, Y.‐Y. , J.‐J. Qian , X.‐L. Hou , et al. 2023. “Diurnal Emission of Herbivore‐Induced (Z)‐3‐Hexenyl Acetate and Allo‐Ocimene Activates Sweet Potato Defense Responses to Sweet Potato Weevils.” Journal of Integrative Agriculture 22, no. 6: 1782–1796. 10.1016/j.jia.2023.02.020.

[ece371175-bib-0080] Yactayo‐Chang, J. P. , H. V. Tang , J. Mendoza , S. A. Christensen , and A. K. Block . 2020. “Plant Defense Chemicals Against Insect Pests.” Agronomy 10, no. 8: 1156. https://www.mdpi.com/2073‐4395/10/8/1156. 10.3390/agronomy10081156.

[ece371175-bib-0081] Yang, L.‐J. , X.‐G. Li , and H.‐X. Liu . 2011. “Herbivore‐Induced Plant Volatiles in the Leaves of *Ziziphus jujuba* From China.” Chemistry of Natural Compounds 47: 820–822.

[ece371175-bib-0082] YonhapNewsAgency . 2021. “Endangered ‘Northern Pipevine’ Found in Seoul's Jungnangcheon.”

[ece371175-bib-0083] Zhang, L. , L. Du , and B. Poovaiah . 2014. “Calcium Signaling and Biotic Defense Responses in Plants.” Plant Signaling & Behavior 9, no. 11: e973818. 10.4161/15592324.2014.973818. 25482778 PMC4623097

